# A bibliometric study on the utilization of lenvatinib in hepatocellular carcinoma (2014–2022)

**DOI:** 10.3389/fphar.2023.1159286

**Published:** 2023-06-14

**Authors:** Cong-Cong Wang, Cai-Yan Yu, Jing Zhang, Rui Wang, Xiang-Shuo Kong

**Affiliations:** ^1^ Department of Oncology, Yantai Yuhuangding Hospital, Yantai, China; ^2^ School of Medicine, Huanghuai University, Zhumadian, Henan, China; ^3^ Department of Respiratory Oncology, Fushan District People’s Hospital, Yantai, China

**Keywords:** bibliometric, lenvatinib, hepatocellular carcinoma, prognosis, immunotherapy

## Abstract

**Background:** The REFLECT phase-III trial has demonstrated the efficacy of lenvatinib in improving the overall survival of advanced hepatocellular carcinoma (HCC) patients, comparable to sorafenib. The rapidly evolving landscape of hepatocellular carcinoma therapy presents new avenues for lenvatinib. This study aims to provide a scientometric analysis of publications and predict research hotspots in this field.

**Methods:** Relevant publications were sourced from the Web of Science Core Collection (WoSCC) database up until November 2022. The bibliometrix tool in R was employed for scientometric analysis and visualization.

**Results:** A total of 879 publications from 2014 to 2022 were obtained from WoSCC that met the established criteria. These studies involved 4,675 researchers from 40 countries, with an average annual growth rate of 102.5%. The highest number of publications was from Japan, followed by China, Italy, and the United States. The largest proportion of studies, 14.0% (*n* = 123), was contributed by FUDAN UNIV. The studies were published in 274 journals, with CANCERS (*n* = 53) being the top journal, followed by FRONTIERS IN ONCOLOGY (*n* = 51) and HEPATOLOGY RESEARCH (*n* = 36). The top ten journals accounted for 31.5% of the 879 studies. The most prolific authors were Kudo M (*n* = 51), Hiraoka A (*n* = 43), and Tsuji K (*n* = 38). A total of 1,333 keywords were analyzed, with the present research hotspots being “immune checkpoint inhibitors,” “prognosis,” and “pd-1.” Co-occurrence clustering analysis revealed the top keywords, authors, publications, and journals. Strong collaboration was identified in the field.

**Conclusion:** This scientometric and visual analysis provides a comprehensive summary of the published articles on lenvatinib in HCC during 2014–2022, highlighting the research hotspots, knowledge domain, and frontiers. The results can provide insights into future research directions in this field.

## 1 Introduction

Hepatocellular carcinoma (HCC) is a prevalent solid tumor that is a major contributor to cancer-related mortality globally (Zhou et al., 2020). Unfortunately, over half of HCC cases are diagnosed at moderate-to-advanced stages (Marrero et al., 2018), which results in poor patient prognoses due to heavy tumor burdens, liver function impairment, and health deterioration, leading to limited treatment options.

Sorafenib, a first-line systemic treatment, was the only therapeutic agent available from 2007 to 2017 and was proven to improve overall survival (OS) compared to placebo in randomized controlled trials (RCTs) (Llovet et al., 2008; Bruix et al., 2017). A phase-III REFLECT trial (Kudo et al., 2018) reported that lenvatinib was as effective as sorafenib in improving OS (13.6 vs. 12.3 months) and superior in improving objective response rate (ORR, 41% vs. 12%) and progression-free survival (PFS, 7.3 vs. 3.6 months) in advanced HCC cases. As a result, international guidelines now recommend lenvatinib as the first-line treatment for advanced HCC.

In recent years, immune checkpoint inhibitors (ICIs) have shown favorable outcomes in HCC treatment. The IMbrave150 study (Finn et al., 2020a) used a combination of atezolizumab and bevacizumab as first-line therapy for advanced HCC and reported improved outcomes such as OS, PFS, disease control rate (DCR), and ORR compared to sorafenib monotherapy. However, the combination of atezolizumab and bevacizumab was not cost-effective prior to a substantial price reduction (Zhang et al., 2021). Lenvatinib is also recommended by international guidelines as a first-line treatment for HCC, but the place of lenvatinib monotherapy or in combination with ICIs in second-line treatment following ICIs has yet to be consistently determined due to insufficient clinical trial data.

Despite the growing number of studies on lenvatinib in HCC, no specific scientometric analysis of its knowledge structure has been conducted. In this study, we conducted the first scientometric analysis of articles on lenvatinib application in HCC, utilizing literature metrological features to evaluate our research outcomes, influence, and cooperation, identify hotspots, and discuss future trends in this field.

## 2 Materials and methods

### 2.1 Data extraction

In the present work, we have employed a rigorous data extraction process to obtain the relevant information on lenvatinib in the context of hepatocellular carcinoma. The Web of Science Core Collection database, a well-respected and high-quality database, was comprehensively searched using the search terms of “lenvatinib” and “hepatocellular carcinoma” up to November 2022. Only articles and reviews published in English that met our eligibility criteria were selected for further analysis, with the “full record and cited references” being the output. Two independent reviewers were involved in this process to ensure the accuracy of the scientometric analysis. The collected data included title, authors, institution, country/region, journal, abstract, keywords, and references, while data papers, book chapters, proceedings papers or meeting abstracts, editorials, duplicates, or unpublished articles were excluded. Any disagreements were resolved through negotiation or consultation with a senior physician.

### 2.2 Statistical methods

For the purpose of scientometric analysis and visualization, we have utilized the R-language package Bibliometrix (Aria and Cuccurullo, 2017). Bibliometrix is a comprehensive and flexible package that provides automatic algorithms and machine intelligence to collect and examine the data. It was used in this work to obtain information on basic data, cited references, trend topics, and landmark literature in the field of lenvatinib in hepatocellular carcinoma, as well as to analyze countries, journals, author productivity, and institutions.

## 3 Results

### 3.1 General features of published study

**FIGURE 1:**
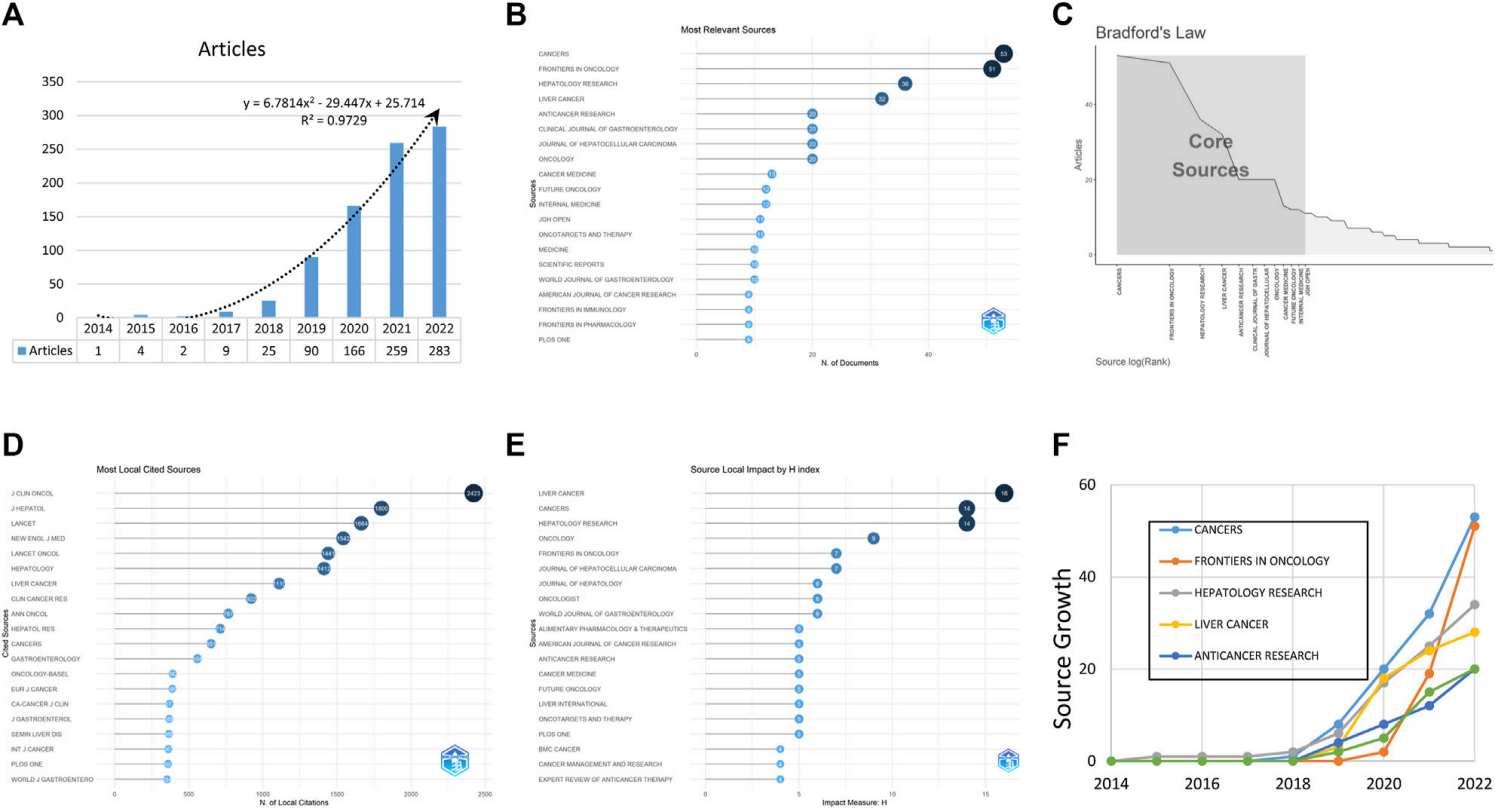
The fundamental data of publications and journals, including the annual scientific study production **(A)**, the most relevant journals **(B)**, the core journals **(C)**, the most locally cited sources **(D)**, the journal impact adjusted by the H index **(E)**, and the journal production over time **(F)**.

In the analysis of published studies on the topic, a total of 879 studies meeting the eligibility criteria were collected in WoSCC. This involved 4,675 authors globally, with an average annual increase rate of 102.5%. The number of published studies increased over time, with a particularly fast increase trend after 2017, accounting for 94.7% of all published studies (Figure 1A).

### 3.2 Analysis of journals

The studies were published in 274 journals, with the highest number in CANCERS (*n* = 53), followed by FRONTIERS IN ONCOLOGY (*n* = 51) and HEPATOLOGY RESEARCH (*n* = 36). The top ten most productive journals accounted for 31.5% of the 879 published studies (Figure 1B) and were identified using Bradford’s law (Figure 1C). Bibliometrix was used to analyze frequently cited sources, with the top three journals being J CLIN ONCOL, J HEPATOL and LANCET (Figure 1D). After adjustment by the H index, the leading journals were LIVER CANCER, CANCERS, and HEPATOLOGY RESEARCH (Figure 1E). The cumulative production of the top five journals over time is displayed in Figure 1F.

### 3.3 Sources of author and institution

**FIGURE 2:**
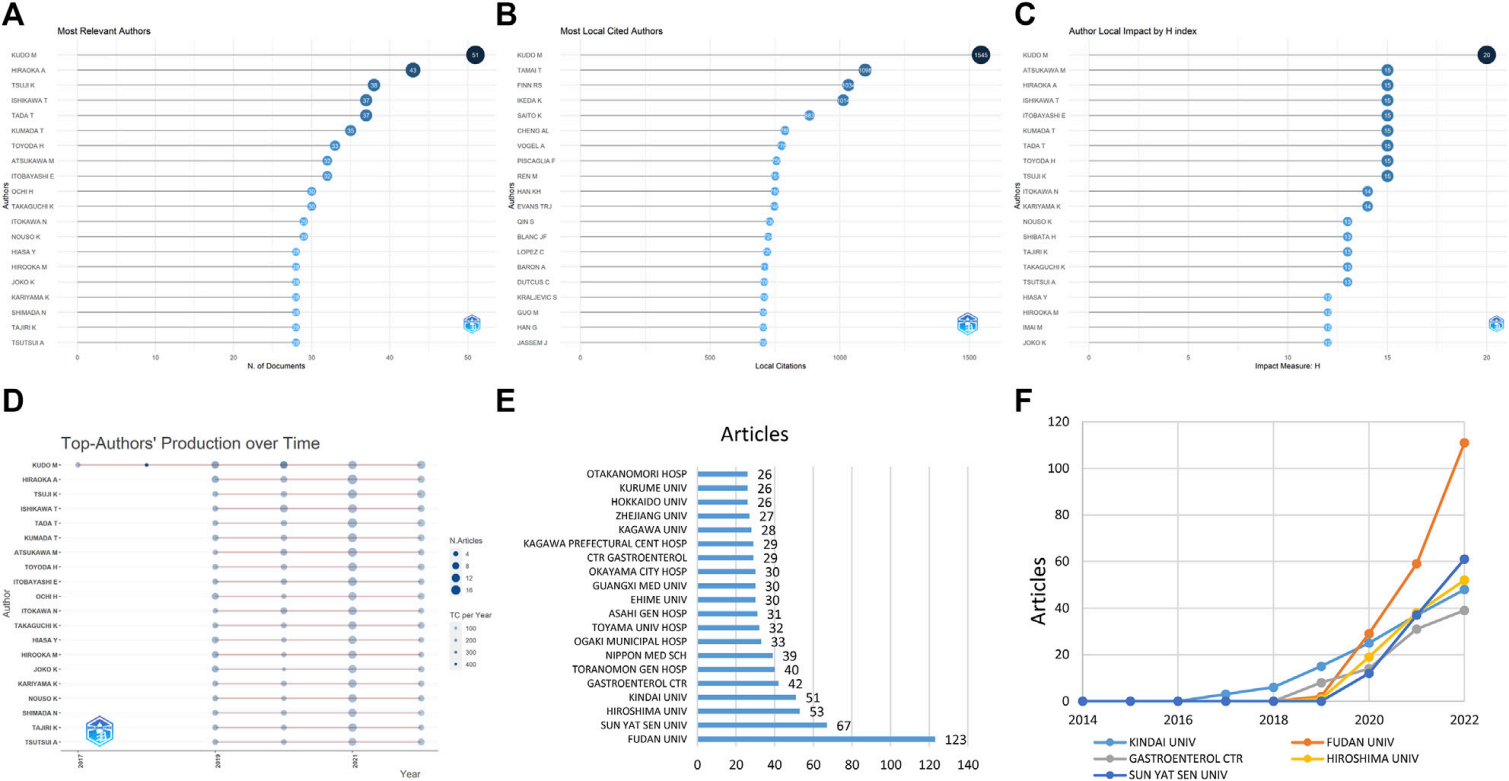
The distributions of authors and institutions. It depicts the most publications by authors **(A)**, the most locally cited authors **(B)**, the author impact adjusted by the H index **(C)**, and the authors with the highest publication number over time **(D)**. The line stands for the timeline of an author from 2017 to 2020 and the color intensity and bubble size positively correspond to total annual citations and document number, respectively. The figure also displays the most publications by institutions **(E)** and the production of institutions over time **(F)**.

Regarding the authors and institutions, there were 5 authors who published over 35 papers, with Kudo M (*n* = 51) being the most productive, followed by Hiraoka A (*n* = 43) and Tsuji K (*n* = 38) (Figure 2A). Kudo M, Tamai T, Finn RS, and Ikeda K were the most frequently cited authors, with more than 1,000 citations (Figure 2B). After adjustment by the H index, Kudo M remained the top author (Figure 2C). The authors’ production over time is shown in Figure 2D, with Kudo M having the longest timeline from 2017 to 2022.

The top 20 institutions producing the most publications are illustrated in Figure 2E, with the top two being from China (FUDAN UNIV and SUN YAT SEN UNIV). FUDAN UNIV was the most productive institution, publishing 123 studies, accounting for 14.0% of the 879 studies (Figure 2F). Although FUDAN UNIV and SUN YAT SEN UNIV in China only started publishing studies on this topic in 2020, they have continued to increase their publication numbers.

### 3.4 Analysis of countries and most cited publications

**FIGURE 3:**
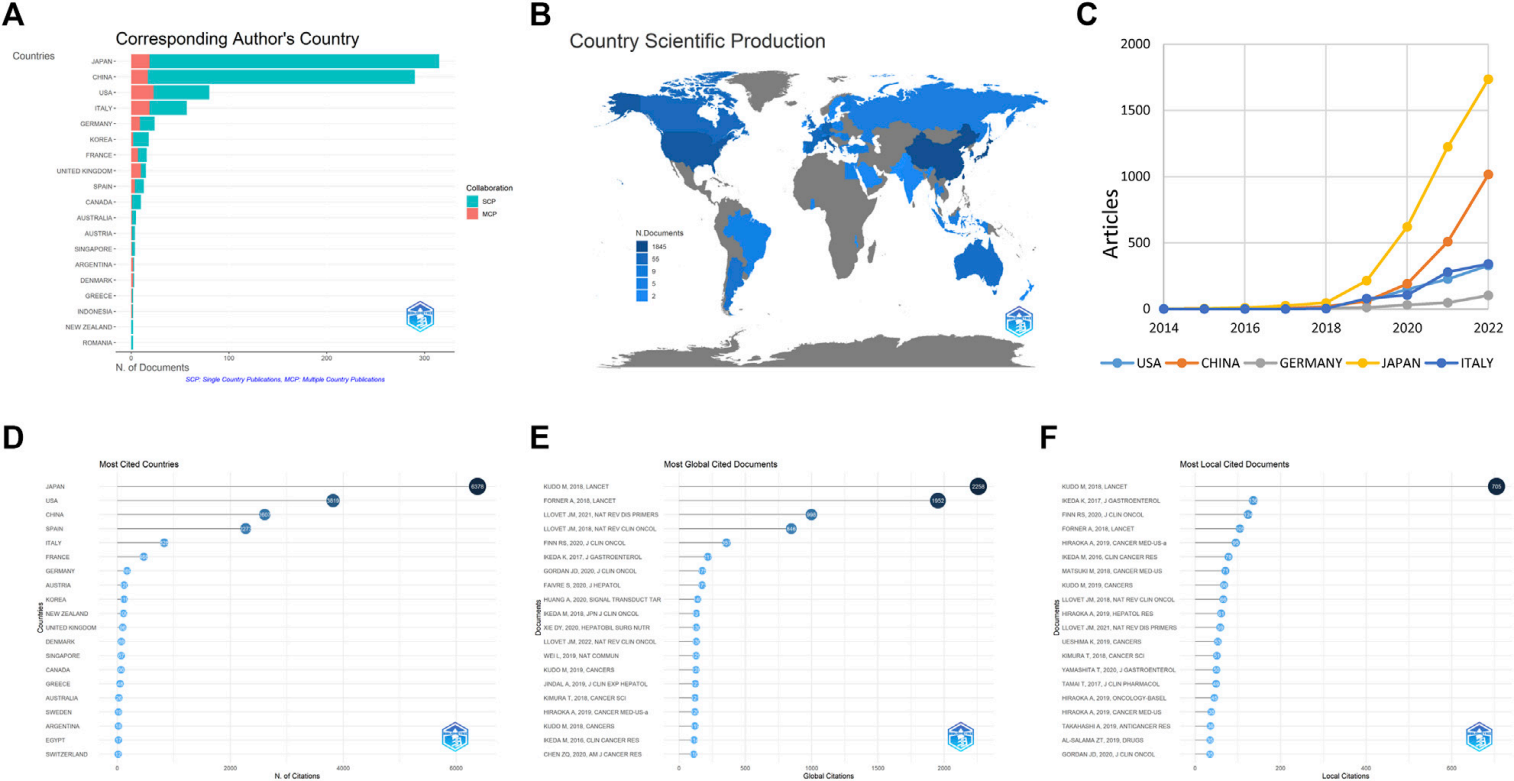
Contributions of different countries, including the top twenty countries with the most articles **(A)**, the production of scientific studies in global countries **(B)**, the top five countries with the greatest number of publications over time **(C)**, the top twenty countries with the greatest citations **(D)**, the top twenty globally cited documents **(E)**, and the top twenty locally cited documents **(F)**. The SCP and MCP refer to single country publications and multiple country publications, respectively.

According to the country of corresponding authors, Japan published the highest number of studies, followed by China and the United States (Figure 3A). The top five countries were in three continents, with two in Asia (Japan and China), one in North America (the United States), and two in Europe (Germany and Italy). Japan had a clear advantage in the number of published studies and an increase in relative number compared to the rest of the countries (Figures 3B, C). Among the top 20 countries with the most publications, Japan also had the highest mean citation rate (Figure 3D). The study by Kudo M published in LANCET in 2018 ranked first in both global (Figure 3E) and local (Figure 3F) citations, indicating its high quality.

### 3.5 Investigation of keyword

**FIGURE 4:**
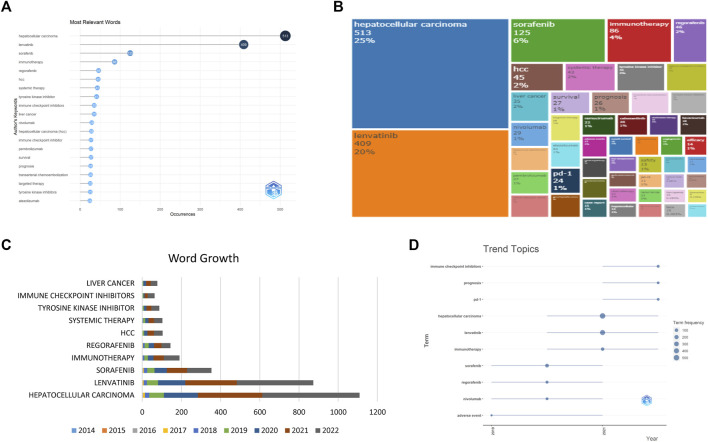
The keyword revolution, including the top keywords with high frequency **(A,B)**, the top ten keywords with the highest accumulation **(C)**, and the trends of the top ten keywords **(D)**.

In our examination of the 879 studies, a total of 1,333 keywords were collected. As depicted in Figures 4A, B, the most frequently used keywords were HCC, lenvatinib, sorafenib, immunotherapy, and regorafenib, demonstrating the significance of these topics in the research. Furthermore, our analysis of the keyword occurrence trend (Figures 4C, D) revealed that the most recent keywords of interest are “ICI”, “prognosis”, and “PD-1", offering insight into potential future research directions.

### 3.6 Analysis of cluster and collaboration network

**FIGURE 5:**
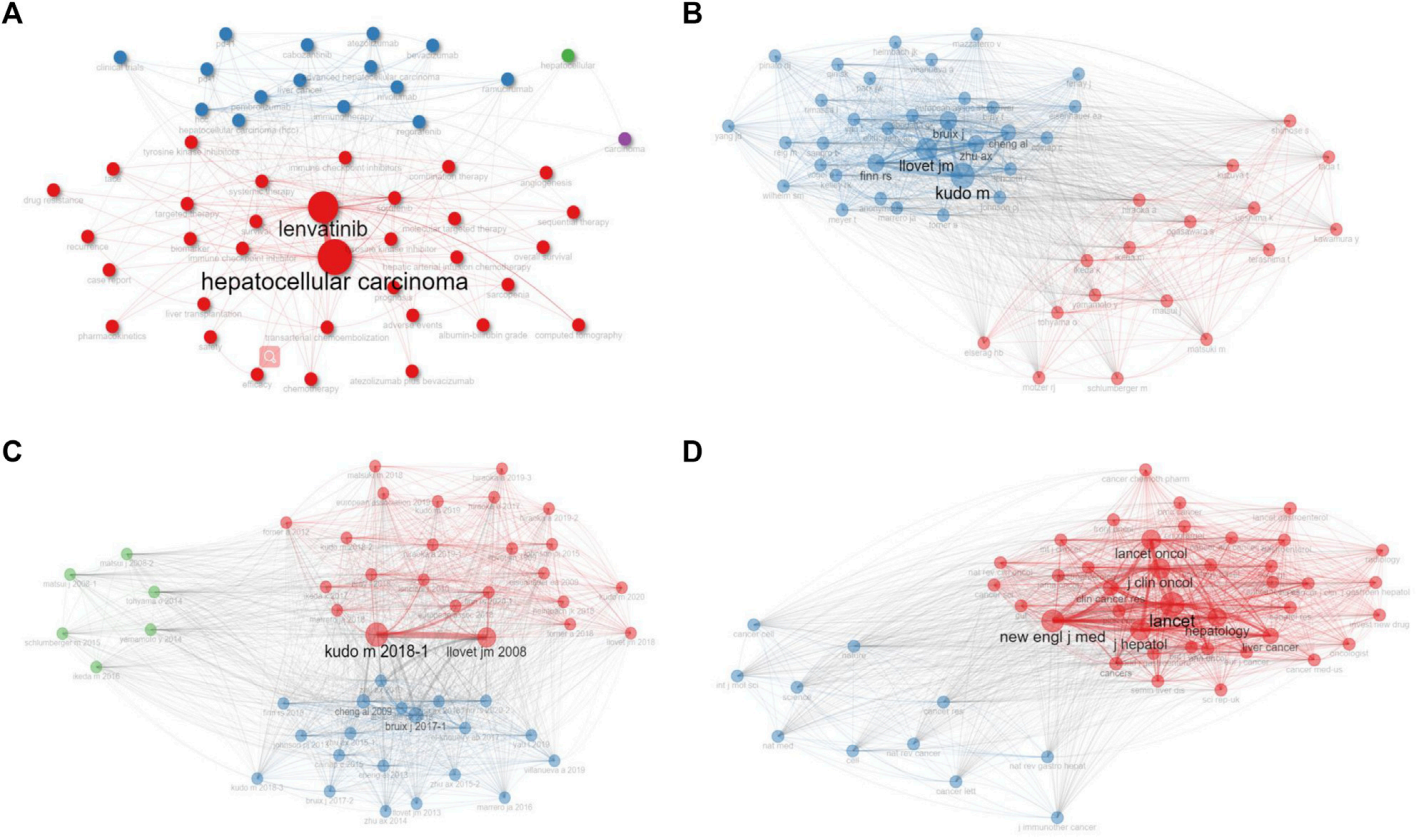
The clustering analysis of keywords **(A)**, authors **(B)**, documents **(C)**, and journals **(D)**, with node size and edge size positively correlated with item occurrence and item co-occurrences, respectively. Nodes with the same colors belong to the same cluster.

Our examination also included a cluster and collaboration network analysis, as represented in Figure 5. These figures display mathematical structures modeling the relationships between the keywords, authors, documents, and journals, where node size and edge size reflect item occurrence and item co-occurrences, respectively. The results indicate that Lenvatinib, Kudo M, Kudo M’s 2018 document, and LANCET hold the highest centrality in their respective clusters.

**FIGURE 6:**
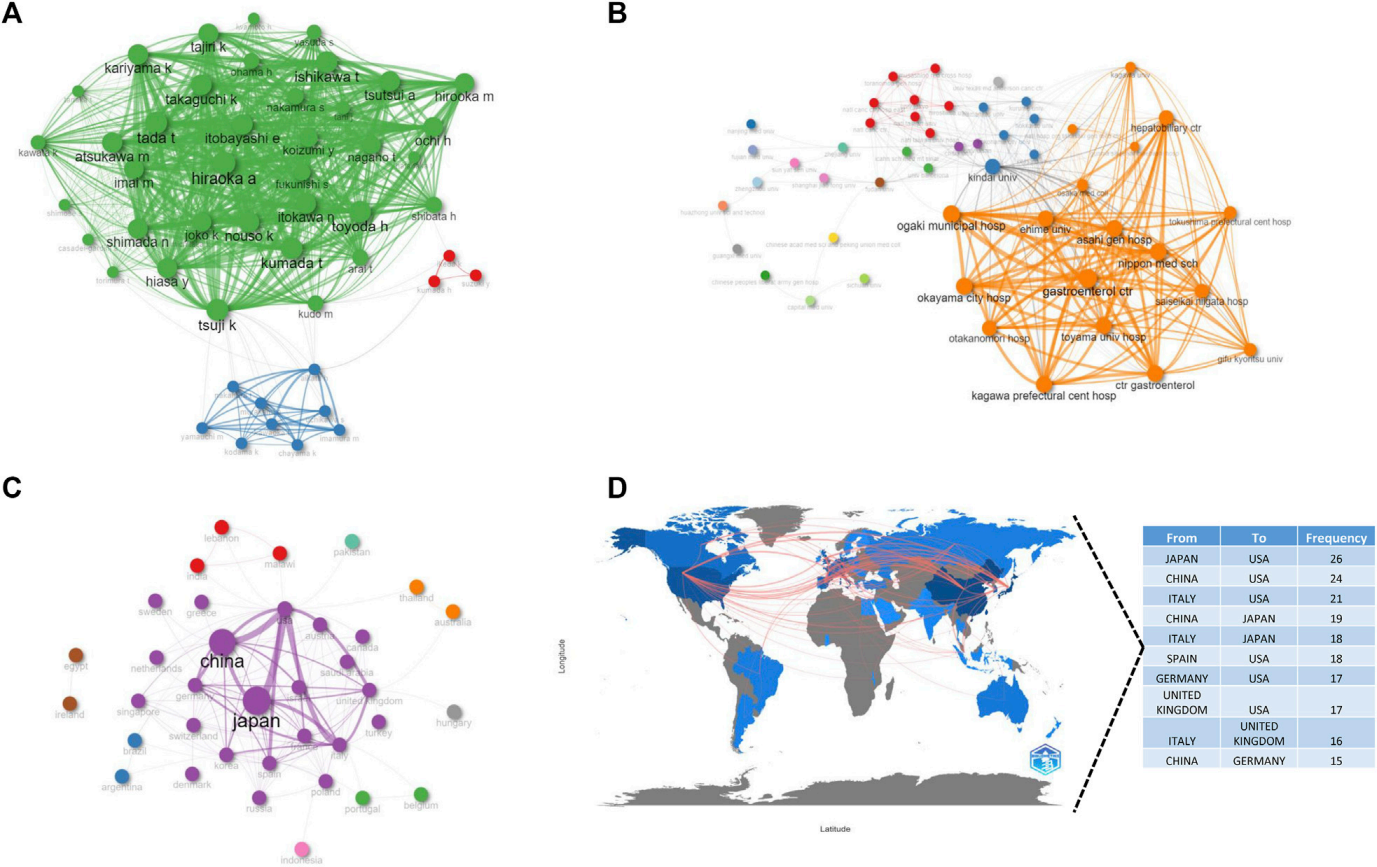
The collaboration network, including the authors **(A)**, institutions **(B)**, countries **(C)**, and the cooperation of countries in contributing to publications **(D)**. Node size and edge size positively correspond to item occurrence and item co-occurrences, respectively, and nodes with the same colors belong to the same cluster.

In addition, we visually depicted international collaboration relationships among authors (Figure 6A), institutions (Figure 6B), and countries (Figures 6C, D). These findings suggest tight cooperation among authors and institutions from Japan, as well as close connections among the top four countries with the most publications, indicating that inter-country collaboration plays a crucial role in research outcome achievement.

## 4 Discussion

In this study, a comprehensive scientometric and visualized analysis was conducted on studies regarding lenvatinib treatment in HCC between 2014 and 2022. Adopting a bibliometric approach provided a more in-depth understanding of research trends and hotspots, and was more objective and thorough compared to traditional methods. A total of 879 published studies were analyzed in this work. The number of publications rapidly increased since 2017, and the most productive countries, institutions, and authors were also identified. According to the keyword distribution analysis, current research hotspots include “ICI,” “prognosis,” and “PD-1.” This analysis provides valuable insight into the evolution of the field, contributing to its development.

Studies on the application of lenvatinib in HCC first appeared in 2014 (Kumar and Huang, 2014), attracting significant attention from researchers. In 2017, the landmark study conducted by Japanese researcher Kudo M and published in LANCET (the REFLECT trial) demonstrated that lenvatinib was as effective as sorafenib in improving OS. The REFLECT trial established critical eligibility criteria, including the absence of surgical indications, moderate-to-advanced stage based on the Barcelona Clinic Liver Cancer system, Child-Pugh class A, and the absence of prior systemic treatment. The results showed that lenvatinib was as effective as sorafenib in improving OS (13.6 vs. 12.3 months; hazard ratio (HR) = 0.92) and had a higher ORR (40.6% and 18.8% by mRECIST and RECIST ver.1.1, separately), reduced time-to-progression (median, 7.4 months), and improved PFS (median, 7.3 months). Following the REFLECT trial, lenvatinib was recommended for moderate-to-advanced stage HCC patients who had progressive disease following transarterial chemoembolization by the American Association for the Study of Liver Diseases in their 2018 guidelines. Additionally, it was also recommended for advanced stage HCC patients and HCC patients with progressive disease or not suitable for locoregional treatments among Child-Pugh class A patients or those with good performance status by the European Association for the Study of the Liver (European Association for the Study of the Liver, 2018). The study captured the critical research by Kudo M, a highly active and renowned author known for his contributions to the exploration of lenvatinib and its underlying mechanisms.

ICIs represent a groundbreaking strategy in the ongoing revolution in cancer treatment. As more studies shed light on the mechanisms by which tumor cells evade immune attack, great attention has been directed towards ICIs (Rizzo et al., 2021). In 2021, the combination of atezolizumab and bevacizumab was recommended as a first-line therapy for unresectable HCC cases due to its superior survival rate compared to sorafenib (Benson et al., 2021). However, as a recent multi-center study suggests, lenvatinib treatment may offer more significant survival benefits when compared to the atezolizumab and bevacizumab combination (Rimini et al., 2022). On the other hand, a phase-III RCT found that anti-PD-1 treatment did not improve survival significantly in advanced HCC cases, as drug resistance was observed in some cases (Finn et al., 2020b). A recent article (Wei et al., 2022) explored the resistance mechanism and suggested a critical role for the PKCa/ZFP64/CSF1 pathway in facilitating immune evasion. Notably, lenvatinib was found to downregulate PKC levels and suppress the PKCa/ZFP64/CSF1 pathway, thus overcoming resistance to anti-PD-1 treatment in HCC, unlike sorafenib. Moreover, a real-world study found that lenvatinib combined with sintilimab produced better long-term results than lenvatinib monotherapy (Zhao et al., 2022). Consequently, lenvatinib holds promise as a monotherapy and in combination with ICIs as a novel treatment option for unresectable HCC cases in clinical practice. It is important to note that combination treatment may result in ICI-related adverse events, and personalized dosing may help mitigate these events while maximizing patient outcomes (Zhao et al., 2022).

Our study provides an overview of the current research landscape and identifies key trends and future prospects in the field of lenvatinib treatment in HCC. Preclinical and clinical research have shown that lenvatinib in combination with ICIs is effective and safe in treating cancers. For example, lenvatinib combined with pembrolizumab has been approved as a second-line treatment for advanced endometrial cancer that has failed systemic treatment (Makker et al., 2020). A phase-1b trial of lenvatinib and pembrolizumab in advanced HCC cases showed favorable antitumor effects with a median OS of 22 months and an acceptable toxicity profile (Finn et al., 2020c). Additionally, the ORR reached 46.0% under mRECIST criteria, with 11 cases achieving complete response and median response duration and PFS of 8.6 and 9.3 months, respectively. In intermediate HCC patients eligible for locoregional treatment, a phase-III trial (LEAP-012) was conducted comparing lenvatinib and pembrolizumab to placebo and TACE (Llovet et al., 2022). The latest published studies shed light on the clinical value of lenvatinib in HCC and provide insight into the current options for systemic treatment (Li et al., 2022; Jiang et al., 2023; Leowattana et al., 2023; Su et al., 2023; Xie et al., 2023; Yang et al., 2023).

Japan, China, Itlay and the United States were the most productive countries, and close collaborations were observed between countries and institutions. However, collaborations between United States and other countries were found to be stronger than those between countries outside United States, implying that international collaborations should be strengthened.

It must be noted that certain limitations exist in our analysis. Firstly, the studies included in this analysis were sourced from only the WoSCC database, potentially causing a biased representation of the data. Secondly, with the recent increase in the number of studies published on the topic, citation counts may not accurately reflect the more recent advancements. Furthermore, alternative bibliometric software utilizing diverse algorithms are available, although the R software employed in our analysis proves to be a powerful tool, it is not without limitations.

In conclusion, this scientometric and visual analysis delves into the data surrounding the application of lenvatinib in HCC, covering the period from 2014 to 2022. Our results offer valuable insights into the treatment of HCC with lenvatinib, serving as a guiding light for future research and the discovery of innovative treatment regimens.

## Data Availability

The original contributions presented in the study are included in the article/supplementary material, further inquiries can be directed to the corresponding authors.
